# Seasonal Water Level Fluctuations Mediate Hydrological Connectivity and Shape Zooplankton Metacommunity in Poyang Lake Floodplain Saucer Lakes

**DOI:** 10.3390/biology15161361

**Published:** 2026-08-10

**Authors:** Xueqing Bian, Qingru Zhang, Zengfei Chen, Jiamin Han, Song Zhang, Ao Zhang, Xianzhe Xu, Haiming Qin

**Affiliations:** School of Life Sciences, Qufu Normal University, Qufu 273165, China; 17861369059@163.com (X.B.);

**Keywords:** zooplankton, metacommunity assembly, water level fluctuation, hydrological connectivity, Poyang Lake

## Abstract

Poyang Lake is a typical floodplain lake hydrologically linked to the middle-lower Yangtze River. Its peripheral saucer-shaped sub-lakes undergo seasonal hydrological alternation between isolation and connectivity driven by annual water level fluctuations. To date, the coupling between seasonal hydrological rhythms and zooplankton metacommunity assembly in these saucer floodplain lakes remains poorly understood. In this study, four representative saucer sub-lakes within the Poyang Lake National Nature Reserve (Banghu, Zhonghuchi, Shahu and Dahuchi) were selected as research objects. Seasonal quantitative zooplankton surveys and synchronous hydro-physicochemical monitoring were conducted across four hydrological phases from 2012 to 2013: low-water isolation (spring), high-water connectivity (summer), water recession transition (autumn), and extreme low-water closure (winter). We systematically explored how water level fluctuations regulate zooplankton metacommunity assembly processes. In total, 95 zooplankton species were identified, among which rotifers (68 species) constituted the absolute dominant taxon, and 15 species were identified as year-round absolute dominants exhibiting regular seasonal succession in response to hydrological shifts. Our results demonstrate that hydrological connectivity plays a strong role in regulating the relative strength of species dispersal and environmental filtering. During spring and winter, complete hydrological isolation blocked inter-lake species exchange, making local environmental filtering the main driver of metacommunity assembly. Isolated sub-lakes harbored fewer total species but abundant endemic taxa, accompanied by pronounced spatial community heterogeneity. When water levels exceeded the 17.3 m threshold in summer, full water exchange between sub-lakes and the main lake coincided with widespread species dispersal, which appeared to represent the primary assembly process. The four sub-lakes shared 23 co-occurring species during this period, with highly homogenized community structures and minimal spatial differentiation (Global test: R = 0.47, *p* = 0.001). In autumn, receding water levels weakened inter-lake connectivity, such that environmental filtering and dispersal jointly structured zooplankton metacommunities; significant spatiotemporal differences were detected in zooplankton density, biomass and α-diversity (*p* < 0.05). Critical hydro-physicochemical factors of environmental filtering included water temperature, chlorophyll a, pH, electrical conductivity and turbidity, yet the dominant limiting factor varied spatially across sub-lakes due to divergent basin topography and water residence time. Species co-occurrence network analysis revealed that zooplankton communities achieved the highest stability during the fully connected summer phase. In early autumn water drawdown, species association networks became fragmented, and community stability reached its annual minimum. This study elucidates the mechanistic link between floodplain lake hydrological rhythms and zooplankton metacommunity assembly, providing theoretical support and baseline data for wetland biodiversity conservation and ecosystem management of Poyang Lake floodplains.

## Simple Summary

Seasonal water level fluctuations alternately connect and disconnect the small surrounding lakes of Poyang Lake from its main water body. However, how such periodic water changes influence tiny aquatic animal communities remains unclear. This study addresses this issue by monitoring seasonal variations of these aquatic communities and water levels between 2012 and 2013. The results show that water connectivity strongly shapes aquatic life communities. At low water levels, isolated lakes develop unique local species and uneven community structures. At high water levels, fully connected waters allow species to spread freely, creating stable and consistent biological communities. Falling water levels in transitional seasons reduce the stability of these aquatic communities. This research explains how water level changes affect aquatic organisms in floodplain lakes. It offers practical support for wetland biodiversity protection and the scientific ecological management of Poyang Lake, effectively maintaining the ecological stability of this vital wetland.

## 1. Introduction

Poyang Lake, China’s largest freshwater lake, lies along the southern bank of the middle-lower Yangtze River (28°24′–29°46′ N, 115°49′–116°46′ E). It functions as a critical flood regulation reservoir for the downstream Yangtze and forms an indispensable component of regional ecological security barriers [1]. Benefiting from its unique geographic location and complex mosaic wetland habitats, Poyang Lake delivers irreplaceable ecosystem services including water purification, biodiversity maintenance, water storage and flood mitigation, underpinning the overall stability of the middle-lower Yangtze aquatic ecosystem [2]. As a seasonally pulsing floodplain lake, Poyang Lake exhibits extreme annual water level fluctuations, which drive predictable seasonal turnover of aquatic biotic communities across the entire lake basin [3,4].

Zooplankton are micro-organisms with short life cycles and high sensitivity to aquatic environmental changes [5]. Habitat and water quality shifts induced by water level fluctuations directly reshape zooplankton community composition and functional traits [6]. As a pivotal intermediate trophic link within lacustrine food webs, zooplankton bridge primary producers and predatory fish, dominating aquatic energy flow and nutrient cycling and serving as the core hub for transferring primary productivity [7]. Ecologically, filter-feeding rotifers and copepods graze phytoplankton and organic detritus, suppressing cyanobacterial bloom formation and providing natural water purification capacity [8]. Meanwhile, zooplankton constitute the primary food resource for fish larvae, adult fishes and overwintering waterbirds; their species composition and abundance directly determine fishery productivity and wetland habitat carrying capacity, sustaining holistic wetland trophic balance [9]. While zooplankton mediate aquatic ecosystem function, ambient water physicochemical conditions conversely constrain zooplankton population dynamics and metacommunity assembly, driving seasonal succession and spatial distribution patterns [10].

Metacommunity ecology posits that spatially separated local communities are interconnected via species dispersal to form integrated regional assemblages, whose assembly is governed by four fundamental processes: environmental filtering, species interactions, dispersal and ecological drift [11,12]. Leibold et al. [13] further formalized four canonical metacommunity paradigms: species sorting (local environments determine species distribution), patch dynamics (habitat patch turnover and dispersal capacity structure communities), mass effects (balanced environmental filtering and inter-patch migration), and neutral theory (community patterns arise from random drift including speciation, extinction and dispersal). Collectively, water physicochemical filtering, species dispersal and interspecific interactions jointly shape zooplankton metacommunity patterns across multi-spatial and temporal scales [14].

Poyang Lake’s seasonal water level dynamics are primarily controlled by flood pulse effects from five inflowing rivers (Gan River, Fu River, Xin River, Rao River, Xiu River), coupled with backwater interference from the Yangtze mainstream, generating pronounced intra-annual water level variability [15]. Such hydrological pulsing drives cyclic succession of aquatic biota throughout the floodplain [3]. Global studies have confirmed divergent dominant metacommunity assembly mechanisms for zooplankton under contrasting hydrodynamic connectivity regimes [16]. International research has extensively documented how climate change and habitat fragmentation restructure coastal and freshwater zooplankton metacommunities, identifying dispersal, suspended solids and temperature as key regulators of species richness and phylogenetic structure [17,18].

Long-term monitoring and manipulative experiments abroad have systematically revealed climate and fragmentation impacts on zooplankton metacommunities in lakes and coastal waters [19]. Experimental evidence further demonstrates rapid local adaptation as a critical regulator of zooplankton assembly, highlighting evolutionary dynamics’ value to metacommunity theory [20]. Regional aquatic surveys indicate that seasonal temperature gradients primarily structure Yellow Sea zooplankton communities, with clear biotic responses to global climatic oscillation [21]. Field manipulations verify that cross-habitat dispersal sustains and elevates zooplankton species richness by replenishing local species pools [22]. Cross-watershed comparisons consistently show that divergent hydrodynamic conditions shift the dominant assembly pathways of zooplankton metacommunities [23]. Existing literature independently clarifies the individual roles of environmental filtering and species dispersal in structuring zooplankton communities. However, few systematic investigations have tracked seasonal shifts in zooplankton metacommunity assembly across complete annual hydrological cycles within seasonally isolated-connected saucer floodplain lakes characterized by extreme water level swings.

To fill this research gap, we selected four hydrologically synchronized saucer-shaped sub-lakes (Banghu, Zhonghuchi, Shahu, Dahuchi) within the Poyang Lake National Nature Reserve for quarterly field sampling in April 2012, July 2012, October 2012 and January 2013. This study addresses three core scientific questions: (1) What spatiotemporal differentiation patterns characterize zooplankton species composition, standing stock and α-diversity across the four saucer sub-lakes? (2) How do dominant zooplankton metacommunity assembly mechanisms shift across distinct hydrological seasons, and what spatial community patterns emerge during each phase? (3) What seasonal succession rules govern zooplankton assemblages under annual water level rise–fall cycles? By resolving these questions, this study aims to unpack the intrinsic mechanisms through which natural hydrological rhythms regulate zooplankton metacommunity formation, advance theoretical understanding of floodplain lake ecosystem structure and function, and provide scientific guidance for wetland conservation and ecological regulation of Poyang Lake and interconnected Yangtze floodplain lakes.

## 2. Materials and Methods

### 2.1. Study Area

Located on the southern bank of the Yangtze River in northern Jiangxi Province, Poyang Lake features prominent seasonal water level variations [24]. The northwest Poyang Lake National Nature Reserve contains nine saucer-shaped sub-lakes, among which Banghu, Zhonghuchi, Shahu and Dahuchi are geographically adjacent and exhibit nearly synchronized hydrological fluctuations. When water levels exceed the connectivity threshold, these sub-lakes merge with the main Poyang Lake, creating uniform water quality across the floodplain; during dry seasons, water levels recede to isolate each sub-lake as independent lentic water bodies with substantial spatial water quality heterogeneity [25].

**Figure 1:**
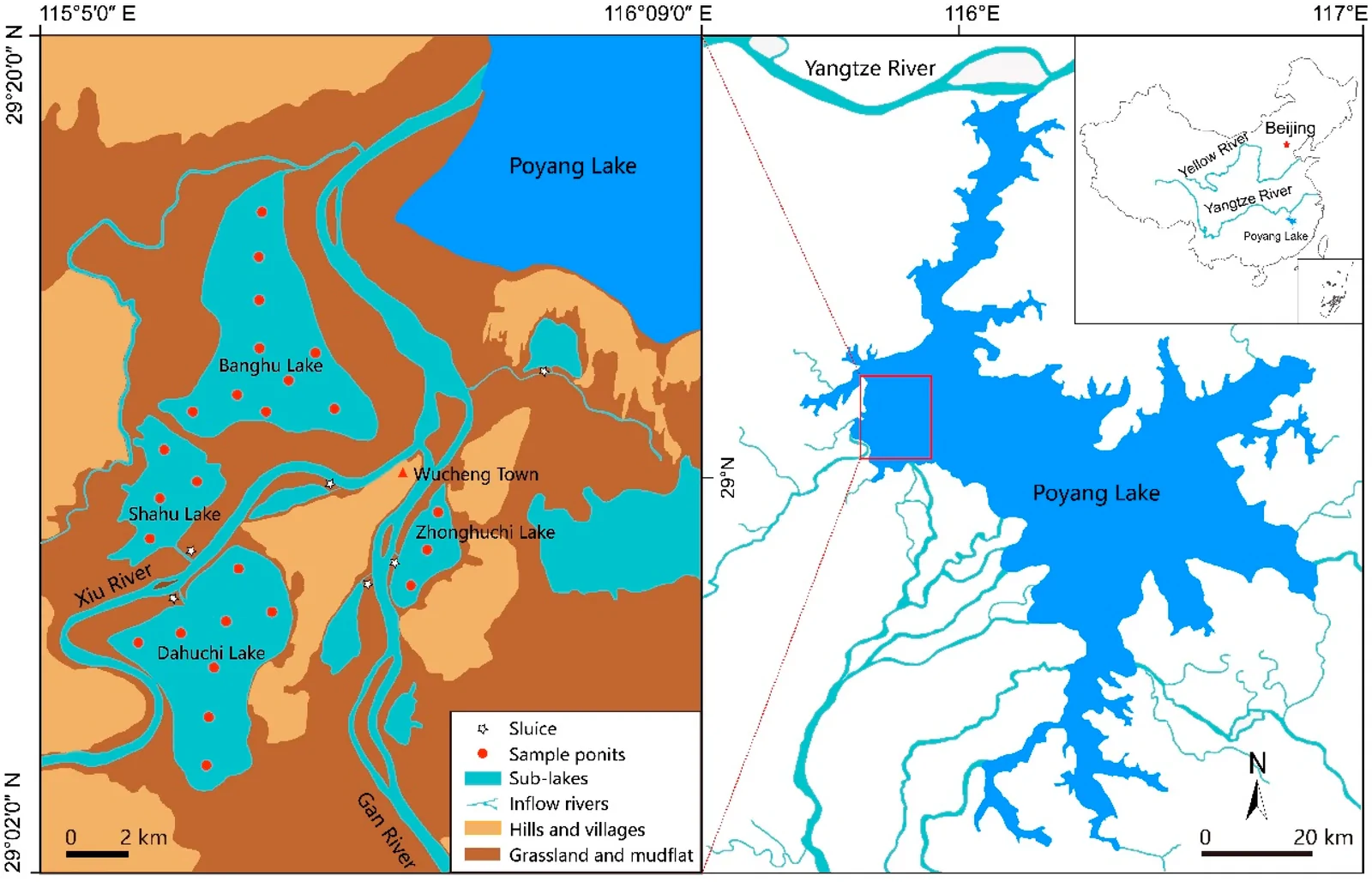
Geographical location of Poyang Lake and distribution of sample points for zooplankton in the four sub-lakes.

Field sampling covered four representative hydrological phases: spring low-water isolation (18–21 April 2012), summer high-water connectivity (21–24 July 2012), autumn water recession transition (15–18 October 2012), and winter extreme low-water closure (15–18 January 2013). Zooplankton quantitative sampling and in situ hydro-physicochemical measurements were conducted synchronously during each campaign. Restrictions apply to the basin’s hydrological monitoring data, and data integration and analysis for this study were only completed after the historical water level archives were declassified and filed. The spatial distribution of sampling sites is illustrated in Figure 1. This dataset acts as a vital ecological baseline prior to the implementation of the Yangtze River Ten-Year Fishing Ban and the frequent regional extreme droughts, fully documenting the original zooplankton community background of Poyang Lake’s saucer-shaped sub-lakes. It offers a critical reference for assessing long-term ecological impacts of human management and climatic disturbances, and bears unique significance for exploring the succession patterns of zooplankton communities in floodplain lakes.

### 2.2. Field Sampling, Species Identification and Water Physicochemical Measurement

#### 2.2.1. Zooplankton Sampling and Taxonomic Identification

Sampling site numbers were stratified according to sub-lake area and shoreline morphology. Spring and summer surveys deployed 10 sites in Banghu, 3 in Zhonghuchi, 4 in Shahu and 8 in Dahuchi. In autumn, Banghu reduced to 9 sites and Dahuchi to 7 sites, while sampling numbers remained unchanged for Zhonghuchi and Shahu. Severe water shrinkage in winter standardized sampling to 3 replicate sites per sub-lake, with three parallel samples collected at each site.

A 5 L water sampler retrieved water at 50 cm depth below the surface. Twenty liters of water were filtered for spring, summer and autumn sampling, while only 2 L were collected in winter due to limited water volume. Samples were concentrated through a 25# plankton net (64 μm mesh size, Pulite Instrument Co., Ltd., Beijing, China), transferred to 50 mL plastic bottles, and fixed in situ with 4% formalin solution (Fucheng Chemical Reagent Co., Ltd., Tianjin, China). In the laboratory, concentrated samples were stained with Rose Bengal sodium salt (Hubei Tuobang Chemical Co., Ltd., Tianmen, China) for 24 h before taxonomic enumeration under an Olympus SZ61 stereomicroscope (Olympus Corporation, Tokyo, Japan). Species identification followed authoritative taxonomic monographs: *Fauna Sinica—Freshwater Cladocera*, *Fauna Sinica—Freshwater Copepoda*, and *Fauna Sinica—Freshwater Rotifera*.

#### 2.2.2. Hydro-Physicochemical Parameter Measurement

In situ multi-parameter water quality analyzers measured water temperature (WT), dissolved oxygen (DO), pH, electrical conductivity (Cond) and turbidity (Turb) at each sampling point. One liter of surface water (50 cm depth) was transported to the laboratory under dark, low-temperature conditions for chlorophyll a (Chl-a) quantification via national standard spectrophotometry (HJ 897-2017) [26].

### 2.3. Data Processing and Statistical Analyses

#### 2.3.1. Dominance and α-Diversity Calculations

The McNaughton dominance index (*Y*) quantified species dominance; taxa with *Y* ≥ 0.1 were defined as absolute dominant species. Three α-diversity metrics were calculated: Shannon–Wiener diversity (*H*′), Margalef richness (*D*), and Pielou evenness (*J*). Calculation formulas:

$$Y=\frac{n_{i}}{N}\cdot f_{i}$$

$$H^{\prime }=-\sum_{i=1}^{s}\frac{n_{i}}{N}\ln\frac{n_{i}}{N}$$

$$D=\frac{S-1}{lnN}$$

$$J=\frac{H^{\prime }}{lnS}$$

where *S* = total zooplankton species richness; *n_i_* = individual count of species *i*; *N* = total zooplankton abundance; *f_i_* = occurrence frequency of species *i*.

#### 2.3.2. Community Ordination and Spatial Variation Analysis

All statistical analyses were performed in R v4.1.2, Statistic 7.0, Primer 5.0, Canoco 4.5 and Gephi 0.10.1. Venn diagrams were generated with the R v4.1.2, Venn Diagram package to visualize shared and endemic species across sub-lakes by season. In the Statistic 7.0 statistical analysis software, one-way ANOVA tested seasonal inter-lake differences in zooplankton density, biomass and α-diversity, with *p* < 0.05 defined as statistically significant. Before conducting the variance analysis, all data were transformed using log (x + 1). Bray–Curtis similarity matrices based on zooplankton abundance were constructed in Primer 5.0 for ANOSIM similarity testing and non-metric multidimensional scaling (NMDS) to visualize spatial community differentiation. Canonical correspondence analysis between zooplankton communities and environmental variables was performed using Canoco 4.5 to clarify their correlations. Detrended correspondence analysis (DCA) preliminarily determined the appropriate ordination model for species-environment relationships: redundancy analysis (RDA) if the first DCA axis eigenvalue < 3; canonical correspondence analysis (CCA) if eigenvalue > 4; both models applicable for eigenvalues 3–4. Monte Carlo permutation tests filtered significant environmental drivers, and biplots visualized species-environment correlations. Species co-occurrence networks were constructed in Gephi 0.10.1. Community stability was quantified by the ratio of network density (D) to average clustering coefficient (T, D/T): lower D/T indicates more stable community structure, while higher D/T signals weaker anti-disturbance capacity.

## 3. Results

### 3.1. Annual Water Level Dynamics of Saucer Sub-Lakes

**Figure 2:**
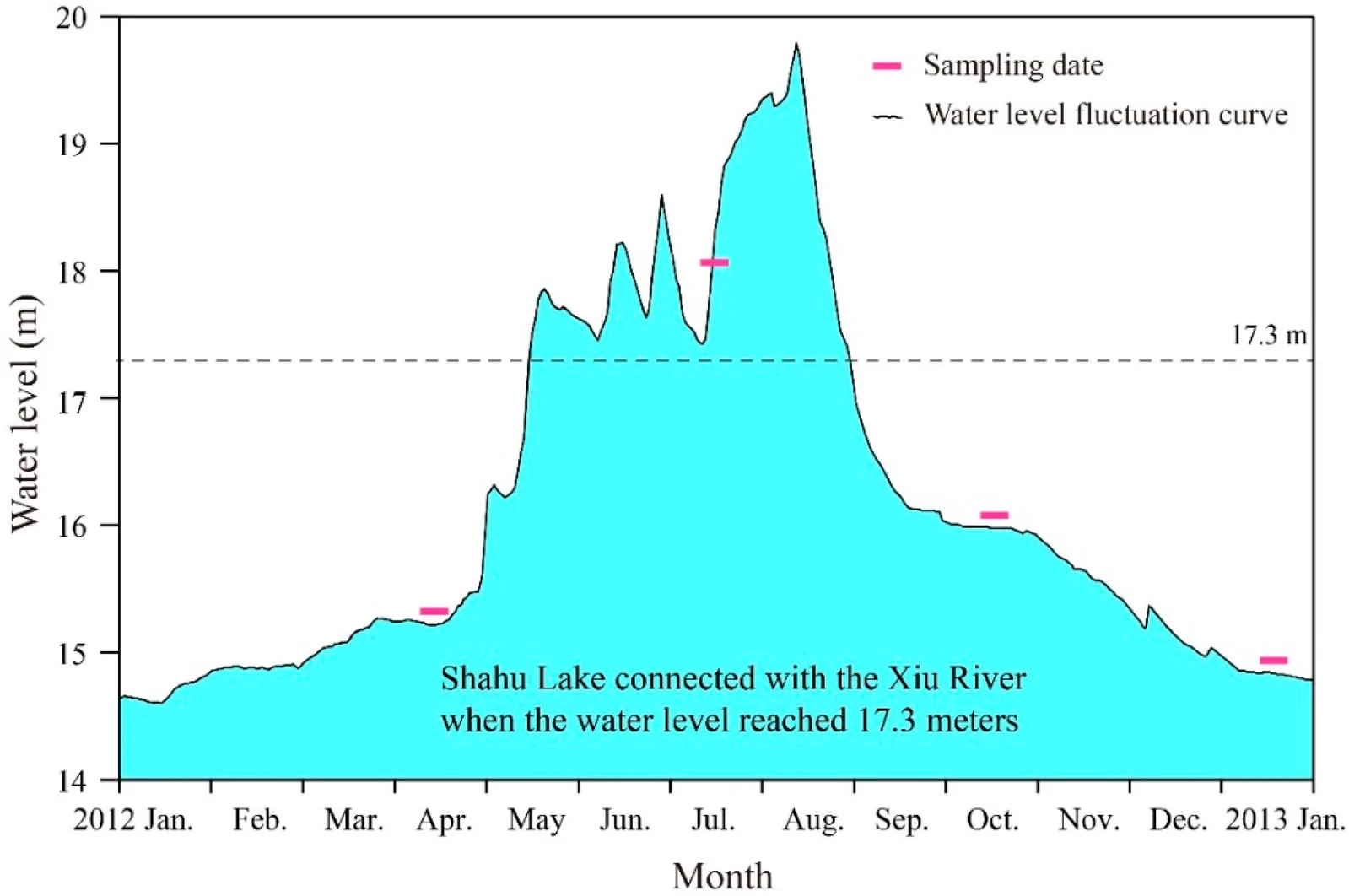
Annual daily variation curve of Shahu Lake water level from 1 January 2012 to 31 January 2013.

Daily water level records of Shahu Lake (1 January 2012–31 January 2013, provided by Poyang Lake National Nature Reserve) represented the unified hydrological rhythm of all four sub-lakes (Figure 2). The 17.3 m water level constituted the critical connectivity threshold: sub-lakes remained isolated from the main lake between January–April and September–December, while full hydraulic connection occurred from May–August when water levels exceeded 17.3 m. The four sampling campaigns fully covered the four representative hydrological phases. Uniform basin elevation across the four sub-lakes ensured synchronized intra-annual water level fluctuations.

### 3.2. Zooplankton Species Composition

**Figure 3:**
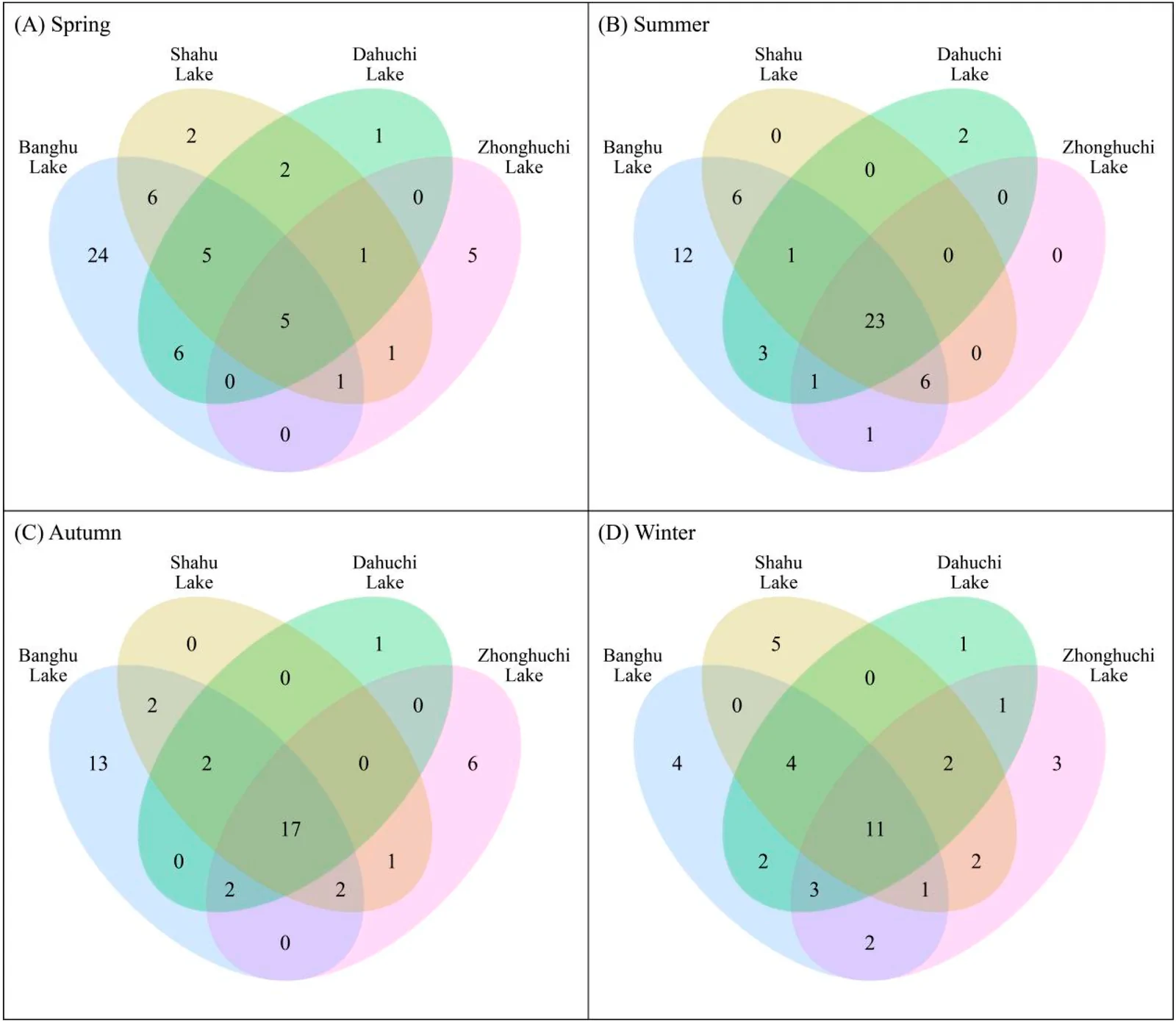
Multiple Venn diagram of zooplankton species in four sub-lakes of Poyang Lake across four seasons.

A total of 95 zooplankton species were recorded throughout the annual survey. Rotifers dominated numerically with 68 species (71.6% of total richness), followed by cladocerans (16 species, 16.8%) and copepods (11 species, 11.6%). Seasonal Venn diagrams revealed clear shifts in shared and endemic species pools (Figure 3). In spring (isolation phase), Only five species co-occurred across all four sub-lakes (*Brachionus budapestiensis*, *Keratella cochlearis*, *Polyarthra vulgaris*, *Daphnia pulex*, *Macrocyclops fuscus*). Banghu harbored 24 endemic species, and one-way ANOVA detected significant inter-lake differences in species richness (*p* < 0.05), demonstrating strong spatial compositional differentiation. In summer (connectivity phase), shared species increased to 23, primarily dominated by *Brachionus* and *Keratella*. Endemic species declined sharply: Banghu contained 12 endemic taxa, Dahuchi only 2, while Shahu and Zhonghuchi had no unique species, with non-significant inter-lake richness differences. In autumn (water recession transition), 17 shared species were observed, with rotifer dominant assemblages consistent with summer. Banghu had 13 endemic species, Zhonghuchi six species, Dahuchi one species, and Shahu none; significant spatial richness heterogeneity persisted (*p* < 0.05). In winter (extreme closure phase), 11 cold-tolerant cosmopolitan rotifers co-occurred across sub-lakes, with endemic taxa detected in all four lakes (Banghu: four, Shahu: five, Zhonghuchi: three, Dahuchi: one). Spatial richness heterogeneity intensified, though ANOVA indicated non-significant inter-lake differences.

### 3.3. Absolute Dominant Species

Fifteen absolute dominant zooplankton species (Y ≥ 0.1) were identified across the sampling period, including seven rotifers, three cladocerans and five copepods (Table 1). Dominant taxa exhibited distinct seasonal turnover patterns. Spring and winter isolation phases lacked universally shared dominant species across all sub-lakes. Cosmopolitan taxa such as *Polyarthra vulgaris*, *Keratella cochlearis* and *Daphnia pulex* only dominated individual sub-lakes, while some species (e.g., *Sinocalanus dorrii*) were exclusively dominant within single sub-lakes. Summer full connectivity homogenized dominant assemblages: pelagic *Polyarthra vulgaris* became a shared dominant across all sub-lakes, alongside thermophilic rotifer blooms of *Brachionus falcatus* and *Asplanchna girodi*. Autumn transitional phase featured the copepod *Microcyclops varicans* as the unified absolute dominant across all four sub-lakes. Several eurythermal taxa (*Keratella cochlearis*, *Asplanchna girodi*) persisted year-round across all sub-lakes, demonstrating broad tolerance to fluctuating water levels and alternating dry–wet habitats.

### 3.4. Spatiotemporal Patterns of Zooplankton Standing Stock

#### 3.4.1. Density

**Figure 4:**
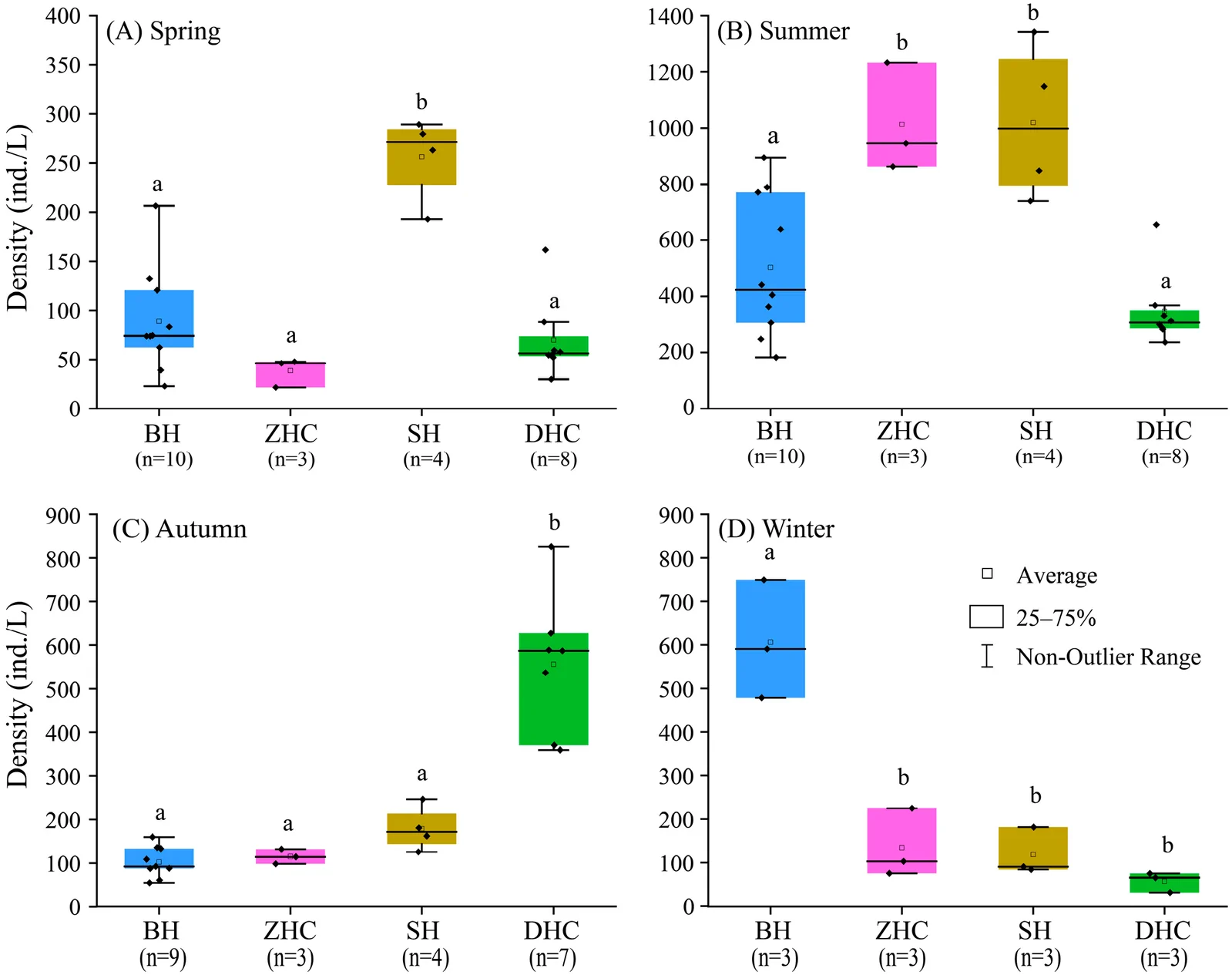
Zooplankton density in four sub-lakes of Poyang Lake each season. Sample size *n* = 3 in winter due to habitat shrinkage. Rhombuses denote zooplankton density at individual sampling sites, and the central horizontal line represents the median zooplankton density of all sampling sites. Different lowercase letters (a, b) indicate significant differences in zooplankton density among the four sub-lakes; groups marked with the same letter have no significant difference.

Across all four sampling seasons, zooplankton density exhibited significant inter-lake discrepancies among the four sub-lakes (*p* < 0.05; Figure 4). In spring, Shahu Lake harbored the highest zooplankton density, reaching 256.27 ± 21.80 ind./L (Figure 4A). During summer, zooplankton densities of Zhonghuchi Lake and Shahu Lake were substantially higher than those of Banghu Lake and Dahuchi Lake, with respective mean values of 1013.54 ± 111.99 ind./L and 1019.61 ± 138.17 ind./L (Figure 4B). In autumn, Dahuchi Lake possessed a significantly greater zooplankton density (556.25 ± 60.31 ind./L) relative to the remaining three sub-lakes (Figure 4C). For winter, Banghu Lake dominated in zooplankton density at 606.22 ± 78.73 ind./L, which was remarkably elevated compared with the other three sub-lakes (Figure 4D).

#### 3.4.2. Biomass

**Figure 5:**
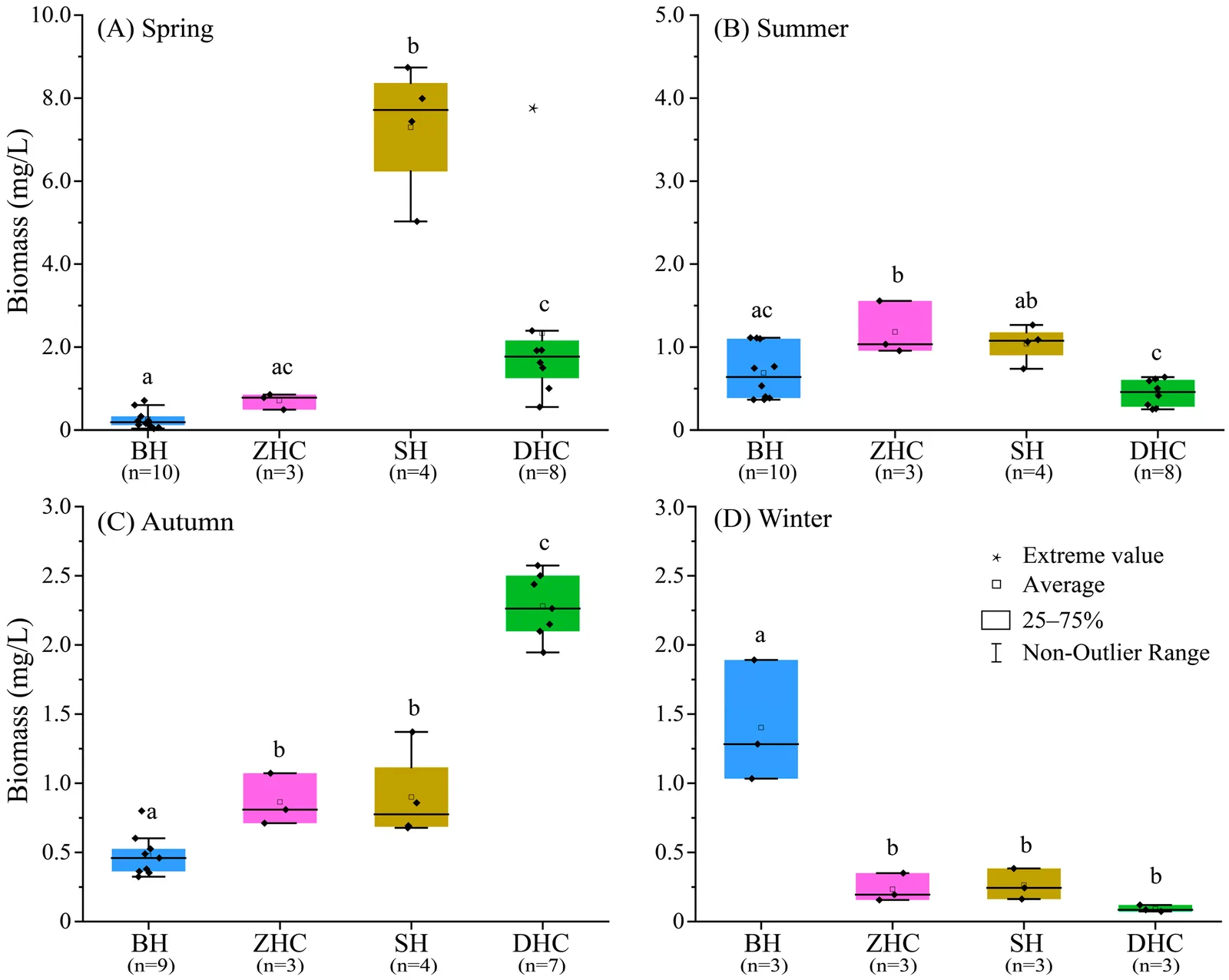
Biomass of zooplankton in the four lakes of Poyang Lake Reserve each season. Sample size *n* = 3 in winter due to habitat shrinkage. Rhombuses denote zooplankton biomass at individual sampling sites, and the central horizontal line represents the median zooplankton biomass of all sampling sites. Different lowercase letters (a, b, c) indicate significant differences in zooplankton biomass among the four sub-lakes; groups marked with the same letter have no significant difference.

The spatial variation of zooplankton biomass largely mirrored that of zooplankton abundance, with significant seasonal and spatial disparities detected across sampling sites (*p* < 0.05; Figure 5). In spring, Shahu Lake exhibited the maximum zooplankton biomass, with an average value of 7.30 ± 0.80 mg/L (Figure 5A). All four sub-lakes maintained relatively low zooplankton biomass levels in summer (Figure 5B). Autumn measurements revealed that Dahuchi Lake had a significantly higher zooplankton biomass (2.28 ± 0.09 mg/L) compared with the other three sub-lakes (Figure 5C). During winter, Banghu Lake recorded a mean zooplankton biomass of 1.40 ± 0.25 mg/L, which was distinctly greater than the biomass values measured in the remaining three sub-lakes (Figure 5D).

### 3.5. Zooplankton α-Diversity

**Figure 6:**
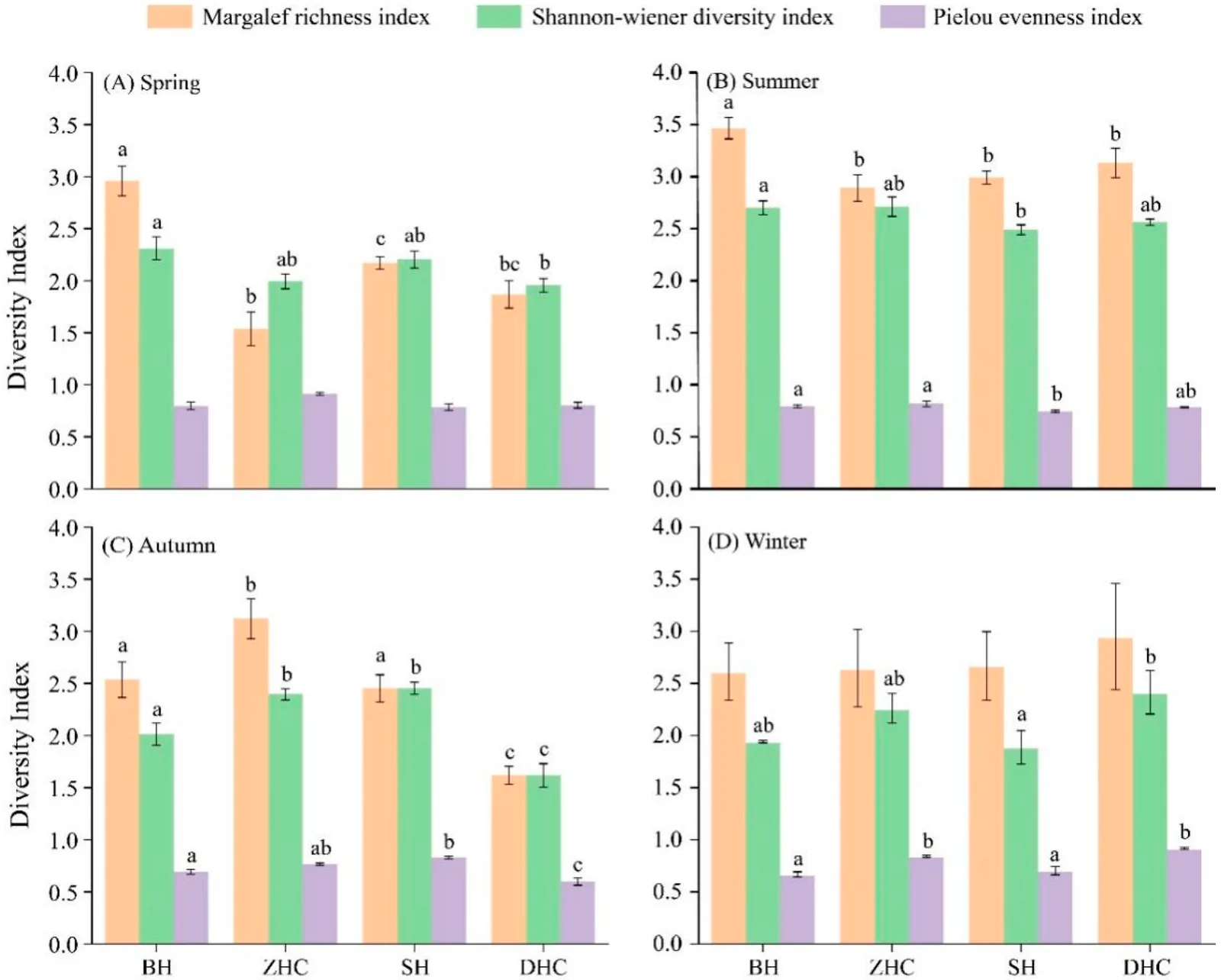
Diversity indices of zooplankton communities in each season. Different lowercase letters (a, b, c) indicate significant differences in zooplankton diversity index among the four sub-lakes; groups marked with the same letter have no significant difference.

Zooplankton Margalef richness index, Shannon–Wiener diversity index and Pielou evenness index all exhibited remarkable spatial variations across the four sampling seasons (Figure 6). In spring, the Margalef and Shannon–Wiener indices differed significantly among sub-lakes (*p* < 0.05), whereas the Pielou evenness index displayed no inter-lake disparity (Figure 6A). During summer, significant inter-lake differences were detected for the Margalef richness and Pielou evenness indices (*p* < 0.05); Banghu Lake attained the maximum Margalef value of 3.47, while the Shannon–Wiener index did not vary spatially across the four sub-lakes (Figure 6B). In autumn, all three biodiversity indices showed significant spatial discrepancies among sub-lakes (*p* < 0.05; Figure 6C). For winter, neither the Margalef index nor the Shannon–Wiener index differed significantly between lakes. By contrast, the Pielou evenness index presented notable inter-lake variation: zooplankton evenness in Zhonghuchi and Dahuchi Lakes was substantially higher than that in Banghu and Shahu Lakes (Figure 6D).

### 3.6. Spatial Structure of Zooplankton Metacommunities

**Figure 7:**
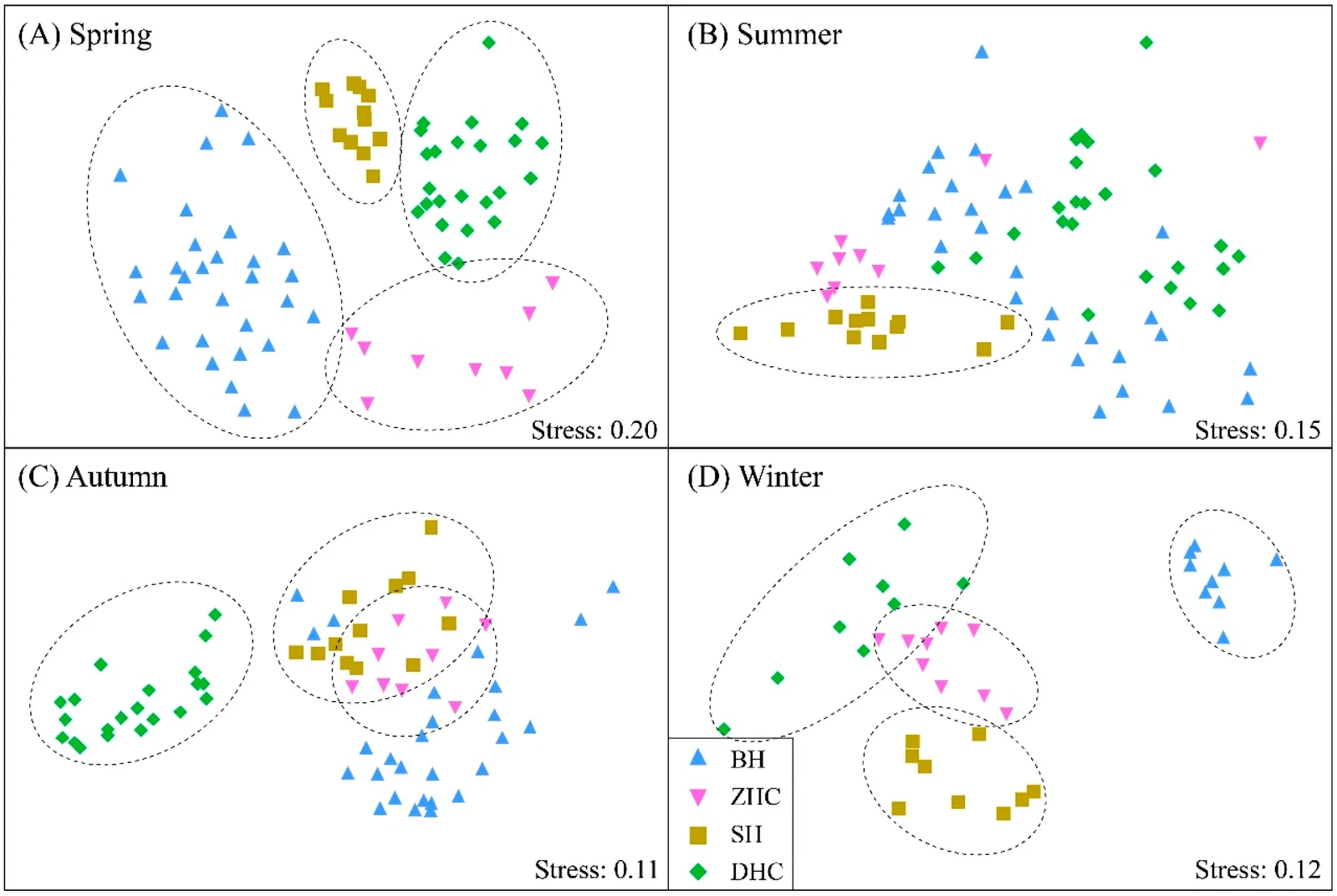
NMDS sorting diagrams of each sub-lake in the Poyang Lake Reserve for the four seasons based on the zooplankton density. Samples enclosed in the same ellipse share similar zooplankton community composition.

Non-metric multidimensional scaling (NMDS) ordination based on zooplankton abundance combined with analysis of similarities (ANOSIM) revealed clear spatial segregation of zooplankton assemblages among the four sub-lakes in spring and winter (Figure 7A,D). ANOSIM tests further verified significant inter-lake differentiation of zooplankton communities during these two seasons (spring: R = 0.802, *p* = 0.001; winter: R = 0.893, *p* = 0.001).

In summer (Figure 7B), overall community similarity across sub-lakes rose substantially, and only the zooplankton assemblage of Shahu Lake was distinctly separated from those of the other three lakes (ANOSIM: R = 0.47, *p* = 0.001). In autumn (Figure 7C), zooplankton assemblages from Banghu, Zhonghuchi and Shahu Lakes displayed divergent community structures; nevertheless, Banghu Lake shared moderate compositional similarity with Zhonghuchi and Shahu Lakes.

### 3.7. Correlations Between Zooplankton and Hydro-Physicochemical Factors

**Figure 8:**
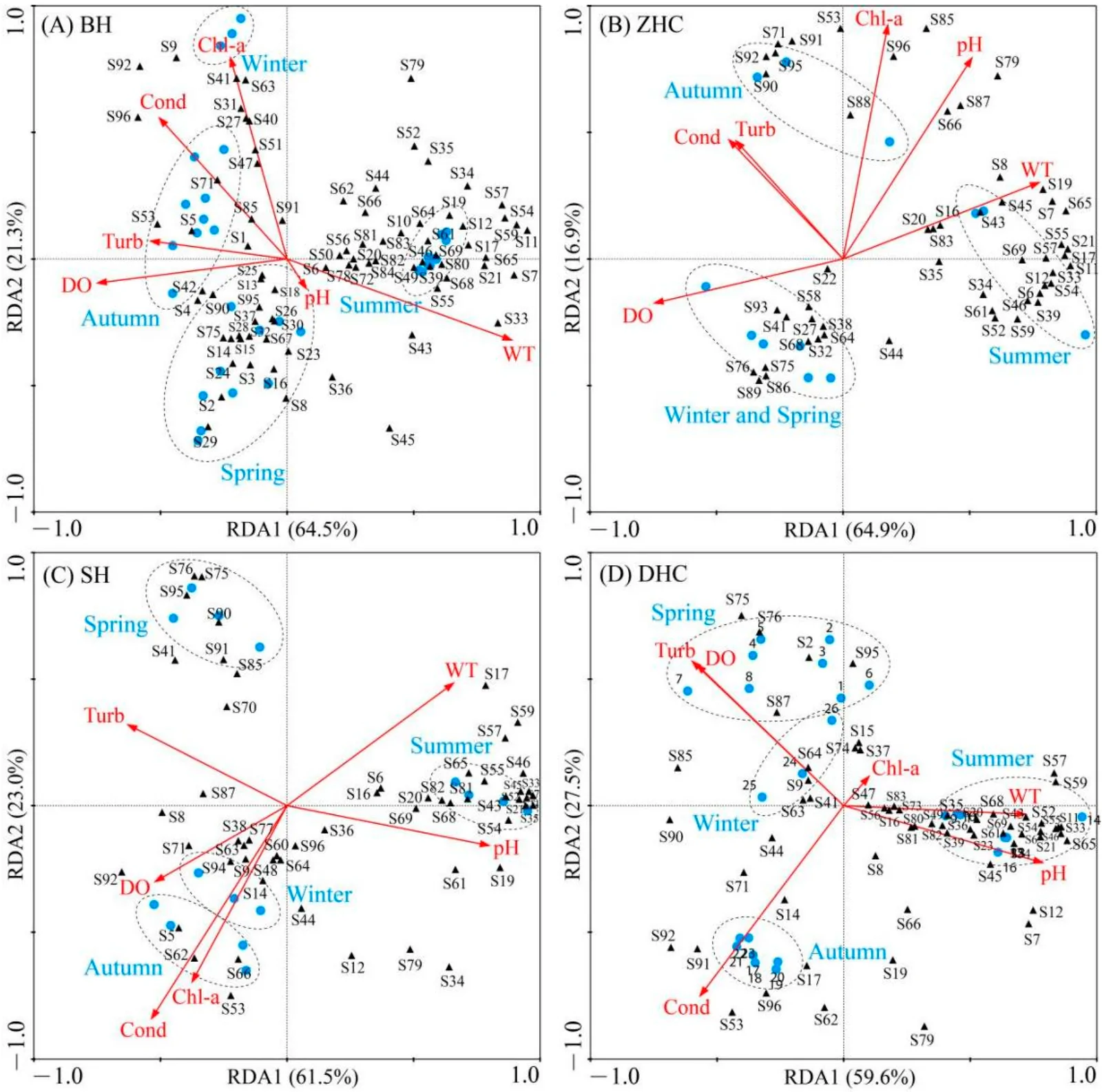
Redundancy analysis ordination plots of zooplankton density and environmental variables in four sub-lakes.

Detrended correspondence analysis (DCA) was independently conducted on zooplankton abundance datasets from the four sub-lakes. Since the eigenvalues of the primary DCA axis across all lakes were less than 4, redundancy analysis (RDA) was subsequently implemented to disentangle the correlations between zooplankton assemblages and environmental factors. RDA ordination indicated that the first two axes collectively explained 81.8–87.1% of the total variation in zooplankton communities across all sub-lakes (Figure 8).

For Banghu Lake, water temperature, chlorophyll a, dissolved oxygen (DO), and conductivity constituted significant drivers structuring zooplankton communities (*p* < 0.05), jointly accounting for 85.8% of the community variance (Figure 8A). In Zhonghuchi Lake, only water temperature imposed a significant regulatory influence on zooplankton community composition (*p* = 0.012), whereas all other physicochemical parameters exerted non-significant impacts; the pair of ordination axes cumulatively captured 64.9% of community variation (Figure 8B). Water temperature, dissolved oxygen, pH, and conductivity were key variables shaping assemblages in Shahu Lake, with the two axes explaining a total of 84.5% of community variance (Figure 8C). In Dahuchi Lake, water temperature, turbidity, pH, and conductivity served as core driving factors, yielding a cumulative explanatory power of 87.1% for community variation (Figure 8D).

Regarding dominant taxa, thermophilic rotifers including *Polyarthra dolichoptera* and *Polyarthra vulgaris* displayed significant positive correlations with water temperature.

### 3.8. Zooplankton Community Stability from Species Co-Occurrence Networks

**Figure 9:**
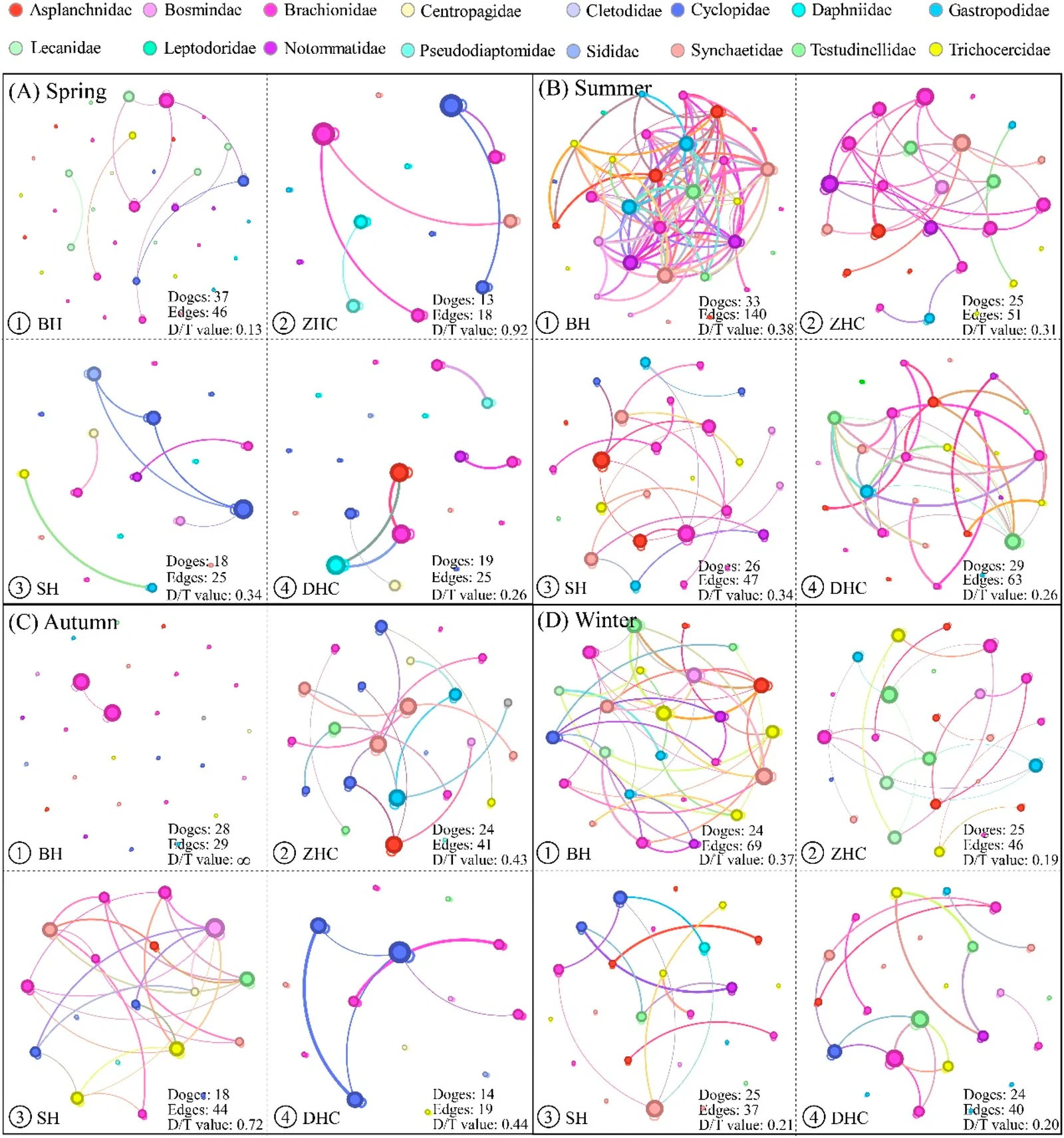
Co-occurrence network analysis based on zooplankton density.

Co-occurrence network analysis illustrated prominent spatial heterogeneity in zooplankton metacommunity stability across the four sub-lakes throughout the sampling campaigns (Figure 9). In spring, Zhonghuchi Lake exhibited the maximum D/T ratio of 0.92, signifying its zooplankton community possessed far weaker stability relative to the other three sub-lakes (Figure 9A). During summer, the D/T values of all sub-lakes stayed at low levels with minor inter-lake discrepancies, which evidenced that zooplankton metacommunities attained their most stable condition in this season (Figure 9B). In autumn, Banghu Lake showed an extreme D/T ratio approaching infinity owing to a zero T value, reflecting that its zooplankton metacommunity suffered the severest structural instability among all study sites (Figure 9C). For winter surveys, D/T indices of the four lakes were generally low, with merely a slight elevation observed in Banghu Lake; this result demonstrated that zooplankton communities maintained robust stability across the whole sub-lake system in winter (Figure 9D).

## 4. Discussion

### 4.1. Seasonal Water Level Fluctuations Mediate the Seasonal Succession of Zooplankton Species and Dominant Taxa

Existing literatures have documented that water level fluctuations remodel aquatic habitat coverage and physicochemical backgrounds, exert persistent filtering pressure on zooplankton assemblages and ultimately trigger seasonal turnover of dominant functional taxa [27]. Consistent with previous findings, the present study reveals that the taxonomic composition, diversity patterns and seasonal shifts of dominant zooplankton across four sub-lakes of Poyang Lake are tightly coupled with lake-wide hydrological rhythms driven by seasonal water level oscillations. Periodic alternation between isolation and hydrological connectivity of sub-lakes from the main lake serves as the pivotal environmental driver structuring annual zooplankton succession. As typical river-connected floodplain lakes along the mid-lower Yangtze River, Poyang’s sub-lakes exhibit regular annual hydrological gradients [28]. Cyclic shifts in hydrological connectivity are closely associated with variations in zooplankton dispersal, habitat utilization and community assembly trajectories, which aligns with the universal “water-level-driven biotic rhythm” ecological paradigm reported for Yangtze floodplain lacustrine systems [29].

Water-level-mediated habitat connectivity is a core determinant of spatial differentiation and seasonal dynamics in aquatic biota [30]. A total of 95 zooplankton species were identified across sampling campaigns, among which rotifers constituted the absolute dominant group, consistent with the community structure trait typical of temperate shallow lakes. Hydrological regime shifts act as key drivers reshaping aquatic species assemblages [31]. During the dry isolation period (spring), sub-lakes were hydrologically disconnected with blocked water exchange, which amplified environmental heterogeneity and intensified habitat filtering [32]. In support of this mechanism, only five zooplankton species were shared by all four sub-lakes in spring, while Banghu Lake harbored up to 24 endemic species. By contrast, summer high-water stages facilitated full hydrological linkage between sub-lakes and the main lake, eliminating habitat barriers and enabling unimpeded material and organismal exchange. Homogenized aquatic habitats substantially attenuated habitat screening effects [33], elevating the number of shared species to 23; Shahu and Zhonghuchi Lake even exhibited no endemic taxa, and inter-lake disparities in species richness vanished entirely. Autumn represented a transitional hydrological phase featuring gradual water recession and connectivity breakdown, yielding intermediate community configurations. The number of shared species declined to 17, and pronounced spatial divergence in species richness re-emerged among sub-lakes [34]. Under the winter extreme drawdown regime, all sub-lakes were fully segregated [35]. Dual filtering pressure imposed by low temperature and stagnant lentic water favored cold-tolerant generalist species, and each sub-lake redeveloped unique endemic assemblages, leading to a rebound in compositional spatial heterogeneity of zooplankton.

Seasonal water level oscillations further govern the turnover of dominant aquatic taxa, as distinct hydrological periods form divergent habitats that select for species with disparate environmental adaptabilities [36]. In this study, seasonal shifts in the dominance of 15 absolute zooplankton taxa exhibited tight synchrony with water level, habitat configuration and thermal regimes. During spring and winter dry phases, sub-lakes featured stagnant narrow water bodies with low temperatures [37], and no pan-lake shared dominant taxa were detected. Widespread taxa including *Polyarthra vulgaris* and *Keratella cochlearis* only dominated isolated individual sub-lakes, while *Sinocalanus dorrii* merely attained dominance in a single lake, corroborating the strong filtering capacity of fragmented lentic microhabitats [38]. In summer, elevated water levels integrated sub-lakes into an extensive open-water system, and rising temperatures generated favorable conditions for thermophilic, highly dispersive zooplankton [39,40]. Accordingly, *Polyarthra vulgaris* became the ubiquitous dominant taxon across all four sub-lakes, while thermophilic rotifers *Brachionus falcatus* and *Asplanchna girodi* bloomed pervasively, demonstrating that homogenized connected habitats homogenize the structure of dominant zooplankton assemblages [41]. During autumn water drawdown, continuous water recession increased habitat complexity, and the copepod *Microcyclops varicans* replaced rotifers as the pan-lake shared dominant species, marking a prominent seasonal turnover of core taxa. Notably, *Keratella cochlearis* and *Asplanchna girodi* occurred persistently across all four seasons in every sub-lake, implying exceptional tolerance to fluctuating water levels and alternating dry–wet environments; these two taxa represent key generalist components within the zooplankton communities of Poyang’s sub-lake system.

### 4.2. Hydrological Connectivity Modulates Zooplankton Standing Stock and Reshapes Spatial Patterns of α Diversity

Hydrological connectivity between sub-lakes and the main water body of Poyang Lake National Nature Reserve is defined by a critical water level threshold of 17.3 m [42]. As water levels fluctuate seasonally, the four investigated sub-lakes undergo four distinct hydrological phases: spring low-water isolation, summer high-water full connectivity, autumn water-retreat transition, and winter extreme drawdown closure (Figure 2). Such periodic hydrological shifts generate four divergent habitat regimes (isolated, connected, transitional, ultra-shallow closed waters), which constitute the primary driver governing seasonal disparities in zooplankton abundance and diversity [43]. Our correlation analyses reveal that hydrological conditions of individual sub-lakes exert significant effects on zooplankton standing stock and α diversity metrics (Figure 4, Figure 5 and Figure 6). Notably, owing to extreme water shrinkage in winter, only three valid sampling replicates were obtained for each sub-lake. A sample size of three reduces the statistical power of one-way ANOVA, which may hinder the detection of subtle inter-group differences. This limitation arises naturally from seasonal habitat contraction in floodplain saucer-shaped lakes. Nevertheless, our findings still reflect real seasonal characteristics of zooplankton communities under natural hydrological regimes. Future investigations can increase sampling sites during dry seasons to improve statistical robustness.

Hydrological linkage facilitates lateral water exchange, redistributing nutrients and accelerating zooplankton dispersal while homogenizing aquatic habitats, thereby mediating seasonal fluctuations in zooplankton standing stock [44]. Previous research has confirmed that connectivity represents a key regulatory factor shaping zooplankton population dynamics [45]. During the spring isolated phase, Shahu Lake exhibited the highest zooplankton density and biomass across all four sub-lakes (Figure 4A and Figure 5A). This pattern can be attributed to nutrient accumulation within enclosed lentic waters under dry conditions, which fuels massive proliferation of small-bodied zooplankton. In summer, full hydrological integration between sub-lakes and the main lake drives intensive water exchange that dilutes ambient nutrient pools [46]. Although connectivity imports allochthonous zooplankton and elevates overall species abundance, persistent hydrodynamic disturbance and nutrient dilution suppress biomass accumulation, leading to a lake-wide decline in zooplankton standing stock [47]. As water levels recede and inter-lake connectivity weakens in autumn, Dahuchi Lake displays markedly higher zooplankton density and biomass relative to adjacent sub-lakes (Figure 4C). The elevated standing stock arises from attenuated water exchange: Dahuchi Lake sits at a slightly higher elevation, retaining substantial nutrients after water recession and supporting rapid recovery of zooplankton populations [35]. Under winter extreme drawdown conditions, Banghu Lake outperforms other sub-lakes in both density and biomass. Banghu features an exceptionally flat terrain and is exempt from autumn dike-based aquaculture disturbances; natural drainage restricts water volume to a narrowly confined zone, concentrating zooplankton and their food resources and yielding far higher standing stock than neighboring lakes.

Numerous prior studies have demonstrated that Margalef richness, Shannon–Wiener diversity and Pielou evenness indices of zooplankton undergo pronounced spatial shifts synchronized with hydrological connectivity cycles [48]. Consistent with this consensus, our results reveal phase-dependent spatial differentiation of α diversity across sub-lakes. In spring’s fully isolated stage, significant inter-lake disparities were only detected in Margalef richness and Shannon–Wiener indices, while Pielou evenness remained comparable among all four lakes (Figure 6A). Enclosed stagnant water impeded cross-lake species exchange, yet zooplankton taxa were distributed evenly within individual isolated basins, resulting in consistent community evenness [49]. During summer full connectivity, Banghu Lake attained the maximum Margalef richness value, and pronounced spatial divergence emerged in Pielou evenness (Figure 6B). Continuous water flow delivers abundant allochthonous zooplankton and enriches local species pools, boosting richness [50]. Meanwhile, persistent hydrodynamic turbulence rearranges the quantitative proportions of different zooplankton functional groups, generating heterogeneous evenness patterns across habitats [51]. The autumn transitional phase with weakened connectivity triggered synchronous spatial differentiation in all three α diversity metrics (Figure 6C). Diminished exogenous species input coupled with intensified local habitat filtering reshaped community composition, driving simultaneous divergence in richness, diversity and evenness among sub-lakes [51]. In winter’s ultra-shallow closed period, sub-lakes exhibited insignificant differences in Margalef richness and Shannon–Wiener diversity, whereas Pielou evenness displayed clear spatial separation (Figure 6D). Extreme low-water environmental filtering eliminates most sensitive taxa, leaving only highly tolerant eurytopic species; consequently, sub-lake zooplankton assemblages converge in overall species composition [52]. Nevertheless, inter-lake discrepancies in basin size, macrophyte coverage and anthropogenic interference create divergent microhabitats, altering relative species abundances and ultimately generating distinct evenness gradients [51].

### 4.3. Hydrological Connectivity Modulates Spatial Patterns of Zooplankton Metacommunities

When spring water levels fell below the 17.3 m connectivity threshold, all peripheral sub-lakes of Poyang Lake were hydrologically disconnected from the main lake. Isolation created independent enclosed lentic basins, with substantial inter-lake disparities in terrain, sediment properties and nutrient accumulation developing across discrete water bodies [53]. Cumulative research has identified seasonal hydrological connectivity as the core hydrological process structuring spatiotemporal divergence of biotic communities in floodplain wetland ecosystems [34]. Annual water level fluctuations trigger cyclic shifts between isolated and connected states among sub-lakes and the main lake; such periodic hydrological alternations serve as the fundamental driver of seasonal succession for lacustrine and wetland aquatic assemblages [39]. Consistent with existing frameworks, our observations demonstrated clear seasonal shifts in zooplankton metacommunity spatial heterogeneity: pronounced inter-lake differentiation prevailed under spring and winter isolation, spatial homogenization emerged during summer full connectivity, while community divergence rebounded in the autumn transitional phase (Figure 7).

In spring, complete hydrological separation eliminated cross-lake water exchange, blocking zooplankton dispersal and facilitating the formation of endemic local assemblages within individual sub-lakes (Figure 7A). By summer, water levels surpassed the critical threshold to integrate sub-lakes and the main lake. Intensive cross-basin water exchange drastically smoothed environmental heterogeneity, and advective transport along hydrological corridors promoted widespread zooplankton dispersal, markedly elevating community similarity across all four sub-lakes (Figure 7B). As water levels receded in autumn, inter-basin connectivity progressively attenuated, restricting material and biotic exchange and amplifying intrinsic environmental disparities among sub-lakes, which gradually restored spatial differentiation of zooplankton metacommunities [54] (Figure 7C). Winter represented the annual drawdown minimum, when all sub-lakes became fully enclosed lentic habitats [55]. Banghu Lake featured a flat terrain without artificial dike fishery disturbance; natural drainage shrank its water volume, concentrating plankton populations and nutrients to form unique extreme dry-season metacommunities (Figure 7D). In contrast, Zhonghuchi, Shahu and Dahuchi Lakes receive water level regulation managed by Poyang Lake National Nature Reserve, retaining stable water depths and developing distinct winter zooplankton assemblages divergent from Banghu Lake (Figure 7D).

Prior ecological theories indicate hydrological connectivity mediates the balance between dispersal filtering and environmental filtering, thereby governing the assembly rules of aquatic metacommunities [56]. Our study further validated this mechanism: summer high-water full connectivity rendered dispersal filtering the dominant assembly process. Unimpeded flow-mediated species exchange homogenized community composition across geographically separated sub-lakes. Conversely, spring rising, autumn receding and winter dry phases featured fragmented, isolated hydrological regimes where environmental filtering prevailed. Limited hydraulic connectivity blocked inter-lake biotic dispersal, and basin-specific physicochemical conditions imposed strong habitat filtering, generating taxonomically divergent zooplankton metacommunities with prominent spatial heterogeneity. Over an annual hydrological cycle, Poyang Lake’s sub-lakes undergo regular alternation between connected and isolated regimes. Such seasonal hydraulic shifts establish a recurring spatial pattern for zooplankton metacommunities: full homogenization in summer, coupled with persistent inter-lake community divergence across spring, autumn and winter.

### 4.4. Hydro-Physicochemical Gradients Drive Environmental Filtering to Structure Zooplankton Metacommunities

Aquatic physicochemical variables act as core media governing environmental filtering of zooplankton assemblages. Seasonal water level oscillations reshape lacustrine environmental backgrounds and impose divergent selective pressures on plankton taxa with divergent physiological tolerances [31]. Within the annual hydrological cycle of Poyang Lake, periodic shifts in inter-sub-lake water exchange intensity concurrently modify multiple key physicochemical parameters, including water temperature, dissolved oxygen (DO), pH, electrical conductivity, chlorophyll a and turbidity, which in turn dominate zooplankton community assembly trajectories [36]. Redundancy analysis (RDA) across all four sub-lakes revealed that the first two canonical axes collectively explained 81.8–87.1% of total variance in zooplankton community composition (Figure 8), demonstrating that the measured hydro-physicochemical variables accounted for most spatial heterogeneity of zooplankton assemblages and validating their central role in mediating environmental filtering and metacommunity divergence.

Consistent with prior research documenting variable filtering strengths under contrasting hydrological regimes [57], Monte Carlo permutation tests in this study identified lake-specific limiting physicochemical drivers (Figure 8). Zooplankton metacommunity assembly in Banghu Lake was co-regulated by water temperature, chlorophyll a, DO and electrical conductivity (Figure 8A). Only water exerted a statistically significant filtering effect on assemblages of Zhonghuchi Lake (*p* = 0.012, Figure 8B). Shahu Lake’s zooplankton communities were constrained by the combined filtering stress of temperature, DO, pH and conductivity, whereas the assembly process of Dahuchi Lake was primarily governed by temperature, turbidity, pH and conductivity. Despite nearly identical elevation and synchronized water level fluctuations across the four sub-lakes that generated analogous connectivity cycles [58], inter-lake physicochemical disparities stemmed from differences in microtopography, hydraulic residence time and internal nutrient mineralization [39].

The functional trait distribution theory posits that environmental gradients directionally filter planktonic taxa with specific temperature tolerances, feeding modes, and life history strategies. This will alter the skewness and kurtosis of the functional trait distribution within the community, ultimately determining the species composition and overall ecological function of the local community [59]. For instance, studies have confirmed that body size of zooplankton, a core functional trait reflecting biomass accumulation and energy metabolic requirements, tends to decrease as water level declines [60]. The results of this study are in line with this pattern. The biomass of zooplankton is significantly lower during the dry season compared to the rainy season. This result directly reflects the directional filtering effect of environmental gradients on the functional traits of zooplankton. Similarly, studies on phytoplankton have shown that dams and sluices alter the natural hydrological regimes of rivers. This leads to an increase in hydrological heterogeneity, which in turn directly affects the distribution and composition of phytoplankton functional groups [61].

The results of RDA ordination in this study further reveal that the key physicochemical factors driving communities in Banghu and Shahu Lake are highly consistent. Both lakes are jointly regulated by water temperature, chlorophyll a and conductivity. In Shahu Lake, the weaker acid-base buffering capacity of the water resulted in an additional significant filtering pressure exerted by pH. The rotifers *Asplanchna priodonta* (S33) and *Asplanchna brightwelli* (S35) are distributed positively along the pH vector and became the dominant species (Figure 8C). This is consistent with the functional trait characteristics of Asplanchna, whose species favor weakly alkaline water. The previously reported conclusion that the hatching rate of resting eggs of *Asplanchna brightwelli* peaks at pH 8.0 further corroborates this result [62]. Meanwhile, the genus *Asplanchna* exhibits morphological plasticity through cyclomorphosis, allowing it to adjust morphological traits such as body size in response to environmental changes. This forms the core functional basis for its adaptation to diverse aquatic environments [63].

The seasonal patterns of zooplankton communities observed in this study also confirm the local filtering effect of physicochemical variables. During summer high-water phases with full hydraulic connectivity, strong hydraulic connectivity throughout the lake facilitates widespread water mixing, which homogenizes environmental gradients including turbidity and conductivity [64]. *Brachionus* relies on its wide thermal tolerance range and becomes the dominant taxa in this period. *Brachionus* represent typical omnivorous filter-feeding zooplankton. They are capable of ingesting diverse particles with sizes between 0.05 and 40 μm. These particles include microalgae like Chlorella, and the rotifers are also able to consume bacteria and microplastic particles. Such a non-selective filter-feeding strategy grants them prominent competitive advantages for resources in habitats with strongly. In addition, the *Brachionus plicatilis* species complex exhibits multiple morphological morphotypes including L-type, SS-type and SM-type, which are adapted to distinct habitats characterized by low temperature and high salinity, high temperature and high salinity, and high temperature and low salinity, respectively. This fully reflects the driving effect of environmental filtering on the diversification of zooplankton functional traits [65]. Other studies have indicated that rising water temperature reduces the complexity of zooplankton functional traits (e.g., body size). Small rotifers may outcompete large copepods to achieve dominance under warm water conditions [66]. This pattern indicates that water temperature acts as the primary sorting factor distinguishing cold-preferring and warm-preferring plankton [67]. It also further confirms that changes in environmental gradients exert a clear directional filtering effect on functional traits of aquatic organisms.

Unlike the well-connected and homogenized hydrological environment in summer, spring, autumn and winter represented hydrologically isolated enclosed stages, where internal nutrient release led to continuous nutrient accumulation. Inter-lake disparities in chlorophyll a, DO, pH and turbidity were greatly amplified, forming a multi-factor synergistic filtering framework that differentiated local zooplankton assemblages across sub-basins.

### 4.5. Water Level Fluctuations Restructure Species Co-Occurrence Networks and Modulate Zooplankton Community Stability

Previous ecological research has validated that the D/T index derived from species co-occurrence networks serves as a quantitative indicator of community resistance against environmental perturbations. Hydrological connectivity and water level-induced habitat stress modify interspecific interactions and resource partitioning, which in turn exert profound impacts on zooplankton community stability [68]. Complementary plankton studies further demonstrated that water-level-mediated connectivity regimes remodel the topological architecture of co-occurrence networks, ultimately regulating community resistance and resilience [69].

The sub-lakes of Poyang Lake follow distinct annual hydrological periodicity. In spring, water levels fall below the connectivity threshold, disconnecting sub-basins from the main lake and forming fragmented lentic habitats with elevated spatial heterogeneity [70]. Under such isolated conditions, Zhonghuchi Lake yielded a D/T value of 0.92, the maximum across all seasonal surveys, which signified the weakest zooplankton community stability (Figure 9A). This pattern stemmed from hydrological isolation that blocked cross-basin dispersal of zooplankton, disrupting balanced interspecific correlations and weakening the network’s capacity to buffer external disturbances [71].

During summer high-water stages, water levels rise to integrate all sub-lakes with the main water body. Intensive hydraulic mixing homogenizes lacustrine microhabitats [72], leading to uniformly low, convergent D/T indices across four sub-lakes. The balanced topological structure of zooplankton co-occurrence networks in this season corresponds to the annual peak of community stability (Figure 9B). Full hydraulic linkage facilitates wide-range zooplankton dispersal and frequent species exchange, fostering abundant mutualistic and compensatory interspecific relationships and substantially boosting the network’s resilience to environmental fluctuations [73].

Autumn represents a transitional drawdown phase with gradual connectivity attenuation. Banghu Lake exhibited an extreme network signature where the T value approached zero, pushing the D/T ratio toward infinity, indicating the most fragmented network topology and the poorest zooplankton stability among all sampling periods (Figure 9C). Rapid water recession drastically contracted aquatic habitat volume, triggering local extirpation of sensitive zooplankton taxa and disassembling inherent interspecific associations within assemblages [74].

In winter, water levels decline to the annual minimum, and all sub-lakes shift to ultra-shallow closed lentic systems [75]. Despite complete isolation, winter hydraulic conditions remain relatively steady with mitigated environmental stress; correspondingly, D/T indices of all sub-lakes stayed at low levels, reflecting intact and stable zooplankton co-occurrence networks. Collectively, seasonal water level oscillations govern periodic shifts in inter-sub-lake connectivity, restructure the pairwise association strength within zooplankton co-occurrence networks, and ultimately determine seasonal variability in community stability. Water level fluctuation is therefore the primary driver modulating zooplankton community stability across Poyang’s sub-lake complex.

## 5. Conclusions

Overall, Poyang’s sub-lake system exhibits predictable annual water level periodicity. Cyclic water level shifts reshape habitat connectivity and spatial heterogeneity, and reconstruct continuous aquatic physicochemical filtering gradients. Such hydrological processes regulate interannual zooplankton dynamics from three complementary dimensions: species spatial distribution, α-diversity differentiation, and seasonal turnover of dominant taxa. The recurring “isolation–connectivity” hydrological alternation constitutes the core ecological mechanism driving seasonal succession and spatial divergence of lacustrine zooplankton assemblages. This study systematically elucidates the tight coupling between annual hydrological rhythms and zooplankton community dynamics, offering robust theoretical support for Poyang wetland biodiversity conservation, rational hydrological regulation and long-term aquatic ecosystem integrity maintenance.

## Figures and Tables

**Table 1:** Absolute dominant zooplankton species in four sub-lakes each season.

Absolute Dominant Species	Dominance (Y)																Species Serial Number
	Spring				Summer				Autumn				Winter				
	BH	ZHC	SH	DHC	BH	ZHC	SH	DHC	BH	ZHC	SH	DHC	BH	ZHC	SH	DHC	
**Rotifera**																	
*Lepadella patella*	0.11	-	-	*	-	-	-	-	*	-	-	-	-	-	-	-	S2
*Brachionus falcatus*	*	-	-	*	0.14	*	*	-	-	-	-	*	*	*	-	*	S11
*Keratella cochlearis*	*	*	*	*	*	*	0.11	*	*	*	*	*	*	*	*	*	S17
*Asplanchna girodi*	-	-	-	-	*	*	*	*	*	*	*	*	*	0.21	*	0.17	S34
*Polyarthra dolichoptera*	*	-	*	*	*	0.17	0.19	*	*	*	*	*	*	*	*	*	S57
*Polyarthra vulgaris*	*	*	*	*	0.18	0.2	0.28	0.25	*	*	*	-	*	0.18	*	0.17	S59
*Synchaeta oblonga*	*	-	-	-	*	*	*	*	*	*	-	*	*	0.14	0.50	*	S61
**Cladocera**																	
*Daphnia pulex*	*	*	0.16	*	-	-	-	*	*	-	-	-	-	-	-	-	S75
*Daphnia hyalina*	-	*	0.10	0.12	-	-	-	-	-	-	-	-	-	-	-	-	S76
*Bosmina longirostris*	*	-	*	-	*	*	*	-	*	0.17	0.19	*	*	*	*	*	S79
**Copepoda**																	
*Sinocalanus dorrii*	*	-	*	0.33	*	*	*	*	-	-	-	-	-	-	-	-	S85
*Limnoithona sinensis*	-	0.12	-	-	-	-	-	*	-	-	-	-	-	-	-	-	S89
*Macrocyclops fuscus*	*	*	0.30	*	*	-	*	-	*	*	*	*	-	-	-	-	S90
*Tropocyclops prasinus*	*	-	*	*	*	-	*	-	*	*	*	0.17	-	-	-	-	S91
*Microcyclops varicans*	*	-	*	*	*	-	-	*	0.20	0.13	0.15	0.51	0.17	*	*	*	S92

Note: “-” means the species were not collected, “*” means the species dominance were less than 0.1. Species Serial Numbers such as S2 and S11 represent the unified serial numbers of all 95 zooplankton species identified in this study, corresponding to the complete species checklist.

## Data Availability

The original contributions presented in the study are included in the article. Further inquiries can be directed to the corresponding author.
